# *Borrelia burgdorferi *stimulation of chemokine secretion by cells of monocyte lineage in patients with Lyme arthritis

**DOI:** 10.1186/ar3128

**Published:** 2010-09-09

**Authors:** Junghee J Shin, Klemen Strle, Lisa J Glickstein, Andrew D Luster, Allen C Steere

**Affiliations:** 1 Center for Immunology and Inflammatory Diseases, Division of Rheumatology, Allergy and Immunology, Massachusetts General Hospital, Harvard Medical School, 55 Fruit Street, Boston, MA 02114, USA

## Abstract

**Introduction:**

Joint fluid in patients with Lyme arthritis often contains high levels of CCL4 and CCL2, which are chemoattractants for monocytes and some T cells, and CXCL9 and CXCL10, which are chemoattractants for CD4+ and CD8+ T effector cells. These chemokines are produced primarily by cells of monocyte lineage in T_H_1-type immune responses. Our goal was to begin to learn how infection with *Borrelia burgdorferi *leads to the secretion of these chemokines, using patient cell samples. We hypothesized that *B. burgdorferi *stimulates chemokine secretion from monocytes/macrophages in multiple ways, thereby linking innate and adaptive immune responses.

**Methods:**

Peripheral blood mononuclear cells (PBMC) from 24 Lyme arthritis patients were stimulated with *B. burgdorferi*, interferon (IFN)-γ, or both, and the levels of CCL4, CCL2, CXCL9 and CXCL10 were measured in culture supernatants. CD14+ monocytes/macrophages from PBMC and synovial fluid mononuclear cells (SFMC) were stimulated in the same way, using available samples. CXCR3, the receptor for CXCL9 and CXCL10, and CCR5, the receptor for CCL4, were assessed on T cells from PBMC and SFMC.

**Results:**

In patients with Lyme arthritis, *B. burgdorferi *but not IFN-γ induced PBMC to secrete CCL4 and CCL2, and *B. burgdorferi *and IFN-γ each stimulated the production of CXCL9 and CXCL10. However, with the CD14+ cell fraction, *B. burgdorferi *alone stimulated the secretion of CCL4; *B. burgdorferi *and IFN-γ together induced CCL2 secretion, and IFN-γ alone stimulated the secretion of CXCL9 and CXCL10. The percentage of T cells expressing CXCR3 or CCR5 was significantly greater in SFMC than PBMC, confirming that T_H_1 effector cells were recruited to inflamed joints. However, when stimulated with *B. burgdorferi *or IFN-γ, SFMC and PBMC responded similarly.

**Conclusions:**

*B. burgdorferi *stimulates PBMC or CD14+ monocytes/macrophages directly to secrete CCL4, but spirochetal stimulation of other intermediate cells, which are present in PBMC, is required to induce CD14+ cells to secrete CCL2, CXCL9 and CXCL10. We conclude that *B. burgdorferi *stimulates monocytes/macrophages directly and indirectly to guide innate and adaptive immune responses in patients with Lyme arthritis.

## Introduction

In the US, Lyme arthritis, which is caused by the tick-borne spirochete *Borrelia burgdorferi*, usually begins with an expanding skin lesion, erythema migrans (EM) [1]. Months later, untreated patients often develop intermittent or persistent arthritis in a few large joints for a period of several years [2]. In EM lesions, perivascular infiltrates of macrophages and CD4^+ ^and CD8^+ ^T cells are found along with small numbers of B cells and plasma cells [3,4]. Similarly, in synovial lesions, macrophages and CD4^+ ^and CD8^+ ^T cells are the primary infiltrating cells, sometimes accompanied by clusters of B cells and plasma cells [5,6]. Thus, cells involved in innate and adaptive immune responses are present at sites of *Borrelia *infection early and late in the illness.

Chemokines (chemotactic cytokines) play a crucial role in the homing of inflammatory cells to infected tissues [7-9]. Early pathogen-induced release of CCL3 and CCL4 by innate immune cells, such as dendritic cells and macrophages, is vital for the initial influx of inflammatory cells [7-9]. Dendritic cells activated by innate stimuli migrate to regional lymph nodes, where they activate the acquired immune system. With T helper 1 (T_H_1)-like immune responses, activated T cells upregulate CXCR3, and macrophage-derived, interferon-gamma (IFN-γ)-inducible chemokines, such as CXCL9 and CXCL10, which are ligands for CXCR3, attract activated T cells into inflamed tissues [7-9]. Thus, chemokines have a critical role in bringing together innate and adaptive immune responses.

Previous studies in Lyme disease clearly show that *B. burgdorferi *induces primarily a T_H_1-type immune response [10-13], leading to the secretion of cytokines and chemokines associated with activation of cells of monocyte lineage. In a study of mRNA expression of 8 cytokines and 12 chemokines in EM skin lesions, there was a predominance of IFN-γ and the IFN-γ-inducible chemokines CCL2, CXCL9, and CXCL10 [4]. Similarly, in a study of the protein levels of 7 cytokines and 7 chemokines in joint fluid in patients with Lyme arthritis, high levels of IFN-γ and CCL2, CCL4, CXCL9, and CXCL10 were found [14]. CCL2 and CCL4 are chemoattractants for monocytes and some T cells, and CXCL9 and CXCL10 are chemoattractants for CD4^+ ^and CD8^+ ^T effector cells [8]. The prominence of these chemokines at sites of infection in Lyme disease correlates well with the types of cells found in infected tissues and fluids [4,14]. However, it is not yet clear how *B. burgdorferi *stimulates the secretion of these chemokines.

In the present study, our goal was to begin to learn how infection with *B. burgdorferi *stimulates monocytes/macrophages to secrete CCL2, CCL4, CXCL9, and CXCL10. For this purpose, we measured the levels of these chemokines in culture supernatants after *B. burgdorferi *or IFN-γ stimulation of peripheral blood mononuclear cells (PBMCs), CD14^+ ^cells from PBMCs, or synovial fluid mononuclear cells (SFMCs) from patients with Lyme arthritis and normal control subjects. We hypothesized that *B. burgdorferi *stimulates chemokine secretion from monocytes/macrophages in multiple ways, thereby linking innate and adaptive immune responses.

## Materials and methods

### Study population

For this study, which was carried out in 2007, frozen PBMCs were tested from 24 Lyme arthritis patients in whom large numbers of cells were available. Concomitant SFMCs were also available in 11 of the 24 patients. In the few patients in whom samples were obtained on more than one date, the first date was used for testing. The 24 patients were seen over a 12-year period, from September 1994 through January 2007. In initial experiments, frozen PBMCs that were collected from 4 normal control subjects from June 2006 through March 2007 were also tested. We used frozen cells from normal subjects so that the cells would be comparable with those from patients.

All patients met the Centers for Disease Control and Prevention criteria for the diagnosis of Lyme disease [15] and were entered into a study called 'Immunity in Lyme Arthritis'. The patients were treated with antibiotic therapy in accordance with the guidelines of the Infectious Diseases Society of America [16]. The Human Investigations Committees at Tufts Medical Center (Boston, MA, USA) (1987 to 2002) and Massachusetts General Hospital (2002 to 2009) approved the study, and all patients (or the parents of patients who were minors) provided written informed consent.

### *B. burgdoferi *or IFN-γ stimulation of PBMCs, CD14^+ ^cells, or SFMCs

On the day of testing, frozen PBMCs (5 × 10^6 ^cells/mL) or SFMCs from patients or normal control subjects were thawed in a 37°C water bath and washed with 50 mL of phosphate-buffered saline (PBS). The viable cells were counted using trypan blue exclusion. The average yields of viable cells were 62% (range of 40% to 90%) from patient samples and 59% (range of 47% to 70%) from normal subjects. With PBMCs, 1 × 10^6 ^cells were set aside for stimulation; in patients in whom enough cells were available, the remaining cells were used for isolation of CD14^+ ^monocytes/macrophages with magnetic-activated cell sorting (MACS) columns (Miltenyi Biotec Inc., Auburn, CA, USA) in accordance with the instructions of the manufacturer. Briefly, cells were suspended in 50 mL of MACS buffer (PBS, 0.5% bovine serum albumin, and 2 mM EDTA [ethylenediaminetetraacetic acid]) and were labeled with primary antigen-presenting cell (APC)-conjugated CD14 antibody (BD Biosciences, San Jose, CA, USA) and incubated with MACS anti-APC microbeads (Miltenyi Biotec Inc.). The CD14^+ ^cells bound with anti-APC microbeads were isolated using a magnetic separation column (Miltenyi Biotec Inc.).

The average yields of CD14^+ ^cells were 11% (range of 3% to 30%) from the PBMCs of patients and 20% (range of 4% to 32%) from those of normal controls. These percentages from frozen cells were similar to the proportion of CD14^+ ^cells (14% ± 3%) recovered from fresh PBMCs in a previous study [17]. In two control subjects in whom sufficient cells were collected, flow cytometric analysis showed that CD14^+ ^cells made up, respectively, 82% and 90% of this cell fraction. Because of limited cell numbers, fluorescence-activated cell sorting (FACS) analysis to determine the proportion of monocytes and macrophages in the CD14^+ ^cell fraction was not done, and CD14^+ ^cells were not isolated from SFMCs.

Whole PBMCs (2 × 10^5 ^cells per well), CD14^+ ^cells from PBMCs (4 × 10^4 ^cells per well), or SFMCs (2 × 10^5 ^cells per well) were suspended in cell culture medium (RPMI, 1% HEPES, 10% human serum, and 1% glutamine) and plated singly in 96-well culture plates (Corning Incorporated, Corning, NY, USA). The number of spirochetes per milliliter of culture was determined using spectrophotometry of spirochetal DNA, as previously described [18]. Live *B. burgdorferi *was used in all experiments.

To determine optimal culture conditions for the stimulation of chemokine secretion, PBMCs from 6 normal control subjects were stimulated with multiplicities of infection (MOIs) ranging from 6 to 500 spirochetes per host cell with durations of incubation ranging from 4 to 120 hours. Although similar results were seen with MOIs from 6 to 100 for all four chemokines, the highest secretion of CCL4 occurred with an MOI of 50. Secretion of CCL4 increased steadily during 5 days in culture, but secretion of CXCL9 and CXCL10 did not appear until the fifth day. This suggested that stimulated cells were viable throughout the 5 days in culture. On the basis of these preliminary findings, cells were incubated with *B. burgdorferi *(strain EM70, an OspC type A strain, MOI of 50), IFN-γ (50 ng), or *B. burgdorferi *and IFN-γ at 37°C in 5% CO_2 _for 5 days.

After incubation, protein levels of CXCL9, CXCL10, CCL2, and CCL4 were determined in cell culture supernatants using cytometric bead array, according to the instructions of the manufacturer (BD Biosciences). Briefly, microbeads precoated with capture antibodies for each chemokine were mixed together in groups and incubated with culture supernatants diluted in sample buffer. Supernatants of PBMCs or SFMCs that had been incubated alone were diluted 5-fold with sample buffer, and cells that had been incubated with *B. burgdorferi *or IFN-γ were diluted 50-fold with this buffer.

### Expression of CXCR3 and CCR5 on T cells in PBMCs or SFMCs

After the collection of supernatants, non-adherent cells were resuspended and collected to determine the expression of CXCR3 or CCR5 and T cells in PBMCs or SFMCs. Harvested cells were washed with FACS buffer (PBS, 1% fetal bovine serum, and 0.1% Na Azide) and stained with the antibodies specific for CD3 (APC; BD Biosciences), CD4 (PerCP; BD Biosciences), CXCR3 (FITC; BD Biosciences), or CCR5 (PE; BD Biosciences). The cell surface expression of these receptors was analyzed by flow cytometry with a FACSCalibur (BD Biosciences).

### Statistics

Secretion of chemokines and chemokine receptor expression were compared between groups by Mann-Whitney rank sum test. Chemokine data were correlated with the length of time that the cells were frozen, using Pearson correlation test. As a correction for multiple variables, *P *values of not more than 0.01 were considered statistically significant.

## Results

### Description of patients with Lyme arthritis

The 24 patients with Lyme arthritis, who were representative of our previously published cohort of 117 patients with this type of arthritis [19], had a median age of 36 years (range of 12 to 67 years); 17 were male and 7 were female. The diagnosis of Lyme arthritis was usually made within 1 to 4 weeks after the onset of knee swelling. Eleven of the 24 patients (46%) were first seen prior to the initiation of antibiotic therapy, 10 were first evaluated during 1- to 4-month courses of oral or intravenous (i.v.) antibiotic treatment, and 3 were not seen until the post-antibiotic period, 7 to 11 months after the start of antibiotics. At their first visit, 13 of the 24 patients (54%) had a positive polymerase chain reaction (PCR) result for *B. burgdorferi *DNA in joint fluid. Of the 13 patients, 6 were seen prior to antibiotic treatment and 7 were evaluated during antibiotic therapy. Seven of the 24 patients had the resolution of arthritis within 3 months after the initiation of antibiotics, defined as antibiotic-responsive arthritis, whereas the remaining 17 patients had persistent arthritis for more than 3 months after at least 8 weeks of oral antibiotics or at least 4 weeks of i.v. antibiotics or both, defined as antibiotic-refractory arthritis [19]. This distribution of refractory and responsive cases is reflective of our role as a referral center.

### Chemokine analysis according to clinical parameters

The chemokine data from cell cultures were compared in patients seen before, during, or after antibiotic therapy, in those with positive or negative PCR results for *B. burgdorferi *DNA in joint fluid, and in those with antibiotic-responsive or antibiotic-refractory arthritis. Although *B. burgdorferi*-stimulated PBMCs from patients with positive PCR results tended to secrete higher levels of CCL2 and CXCL9 in culture than PBMCs from patients with negative PCR results, none of the differences between the groups was statistically significant. In addition, since patients' cells had been stored for as long as 12 years, the chemokine data were analyzed according to the length of time that the cells had been frozen, but no correlation was found. Because timing of cell collection, PCR results, and duration of freezing did not result in differences in chemokine secretion *ex vivo*, the data from all 24 patients are presented together here.

### Chemokine secretion by *B. burgdorferi*- or IFN-γ-stimulated PBMCs and CD14^+ ^monocytes/macrophages from normal control subjects

**Figure 1:**
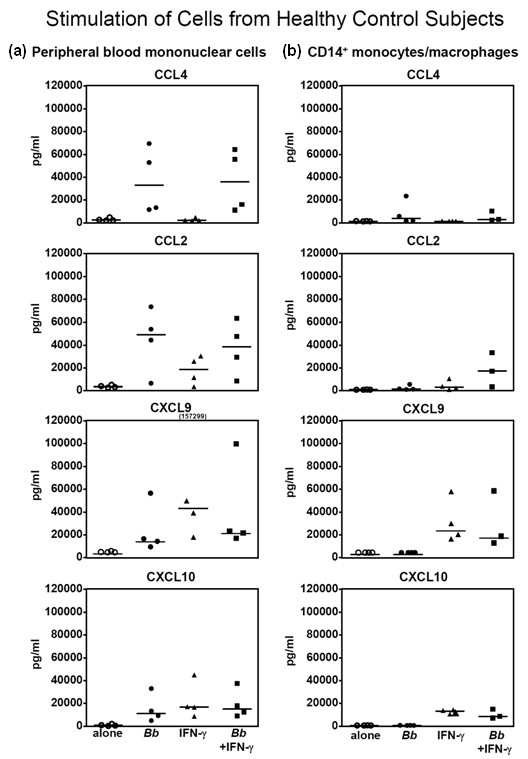
**Secretion of chemokines by peripheral blood mononuclear cells (a) or CD14^+ ^monocytes/macrophages (b) from four normal control subjects**. Cells were not stimulated, or they were stimulated with *Borrelia burgdorferi *or interferon-gamma (IFN-γ) or both. After 5 days of incubation, the levels of chemokines were measured in the culture supernatants. Each point represents the value of an individual control subject. The horizontal bars represent the median values. The highest value for CXCL9 was off the scale and is shown numerically. None of the differences between groups was statistically significant.

In initial experiments, PBMCs were first tested from 4 healthy control subjects from the laboratory. Unstimulated PBMCs from control subjects secreted only basal levels of CCL4, CCL2, CXCL9, or CXCL10 during 5 days in culture (Figure 1a). With *B. burgdorferi *stimulation, the levels of each of the four chemokines tended to be higher; with IFN-γ stimulation, the levels of CCL2, CXCL9, and CXCL10 tended to be greater, and there was no additive effect from stimulation with *B. burgdorferi *and IFN-γ together. However, differences in chemokine secretion among stimulated or unstimulated cells were not statistically significant.

As with PBMCs, unstimulated CD14^+ ^monocytes/macrophages secreted only basal levels of the four chemokines in culture (Figure 1b). In several cases, *B. burgdorferi *alone induced CD14^+ ^cells to secrete small amounts of CCL4; *B. burgdorferi *and IFN-γ together stimulated CCL2 secretion, and IFN-γ seemed to induce CXCL9 and CXCL10 secretion. However, as with PBMCs, the differences between stimulated and unstimulated cells were not statistically significant (Figure 1a, b).

### Chemokine secretion by *B. burgdorferi*- or IFN-γ-stimulated PBMCs and CD14^+ ^monocytes/macrophages from patients with Lyme arthritis

The patterns of chemokine secretion from PBMCs or CD14^+ ^monocytes/macrophages from the 4 normal control subjects were similar to those from the 24 patients with Lyme arthritis. However, the larger number of patients tested allowed a clearer picture of the differences between *B. burgdorferi*- or IFN-γ-stimulated cells.

**Figure 2:**
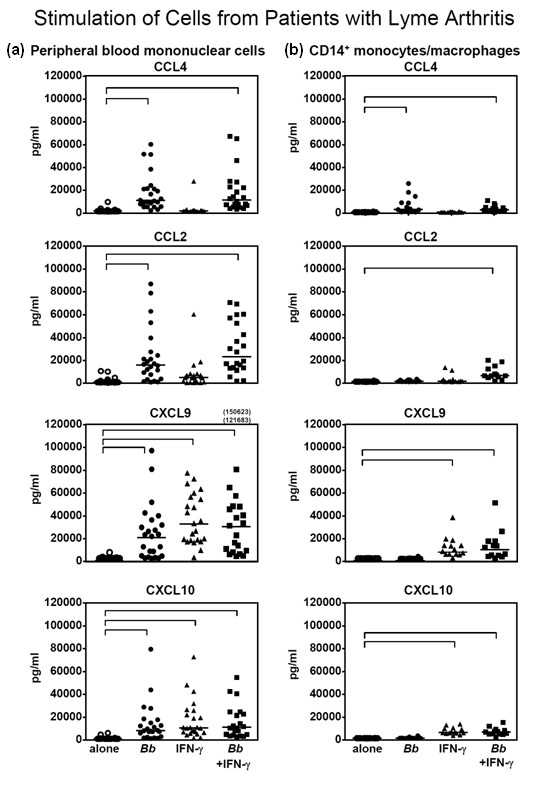
**Secretion of chemokines by peripheral blood mononuclear cells (a) or CD14^+ ^monocytes/macrophages (b) from 24 patients with Lyme arthritis**. Cells were not stimulated, or they were stimulated with *Borrelia burgdorferi *or interferon-gamma (IFN-γ) or both. After 5 days of incubation, the levels of chemokines were measured in the culture supernatants. Each point represents the value of an individual patient. The horizontal bars represent the median values. The highest values for CXCL9 were off the scale and are shown numerically. All significant differences among stimulated and unstimulated cells are shown in brackets and had a *P *value of less than 0.001.

Unstimulated PBMCs from the 24 patients secreted basal levels of CXCL9, CXCL10, CCL2, or CCL4 during 5 days in culture, except in rare cases (Figure 2a). In comparison, *B. burgdorferi *stimulation of PBMCs resulted in the secretion of CCL4 and CCL2, and *B. burgdorferi *and IFN-γ each stimulated the production of CXCL9 and CXCL10. These differences among stimulated and unstimulated cells were statistically significant (*P *< 0.001) (Figure 2a).

In 14 of the 24 patients, large enough numbers of PBMCs were available to separate a fraction containing CD14^+ ^monocytes/macrophages. With the CD14^+ ^cell fraction, *B. burgdorferi *alone stimulated the secretion of CCL4; *B. burgdorferi *and IFN-γ together induced CCL2 secretion, and IFN-γ alone stimulated the secretion of CXCL9 and CXCL10 with no additive effect from *B. burgdorferi *and IFN-γ together (Figure 2b). These differences among stimulated and unstimulated cells were statistically significant (*P *< 0.001) (Figure 2b).

### Chemokine secretion by *B. burgdorferi*- or IFN-γ-stimulated PBMCs and SFMCs

**Figure 3:**
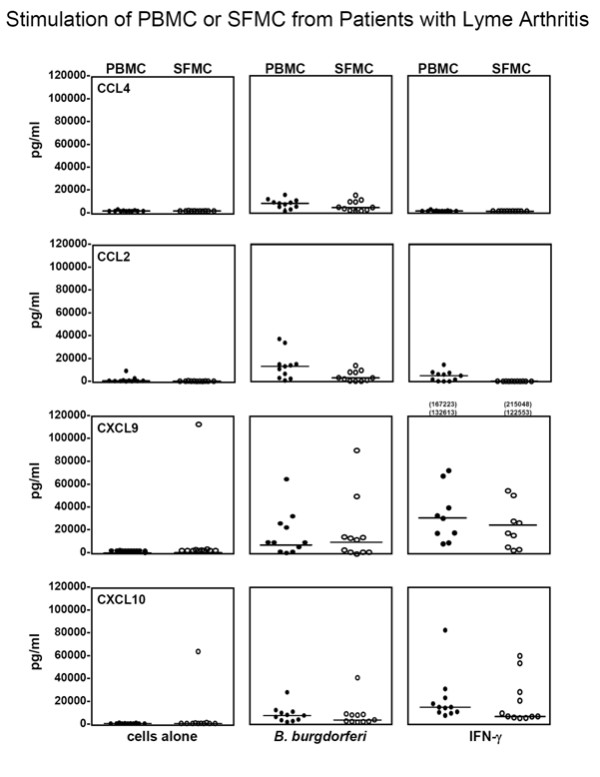
**The secretion of chemokines secreted by unstimulated or *Borrelia burgdorferi*- or interferon-gamma (IFN-γ)-stimulated peripheral blood mononuclear cells (PBMCs) or synovial fluid mononuclear cells (SFMCs) from 11 patients with Lyme arthritis**. After 5 days of incubation, the levels of chemokines were measured in the cell supernatants. The horizontal bars represent the median values. The highest values for CXCL9 were off the scale and are shown numerically. With each stimulus, chemokine levels did not differ significantly between PBMCs and SFMCs.

Since the types of cells, their numbers, and their activation state would presumably be different in SFMCs than PBMCs, cells from these two sites might respond differently to stimuli. For this analysis, concomitant PBMCs and SFMCs were available in 11 of the 24 patients. Owing to limited numbers of cells, CD14^+ ^cells were not separated from SFMCs. Without stimulation, SFMCs or PBMCs secreted basal levels of CXCL9, CXCL10, CCL2, and CCL4, except in one case (Figure 3). *B. burgdorferi*, but not IFN-γ, induced the secretion of CCL4 and CCL2 from PBMCs and SFMCs, whereas *B. burgdorferi *or IFN-γ each induced cells from either compartment to secrete CXCL9 and CXCL10. There were no significant differences between PBMCs and SFMCs in the secretion of these chemokines.

### CXCR3 and CCR5 expression on T cells in PBMCs and SFMCs

**Figure 4:**
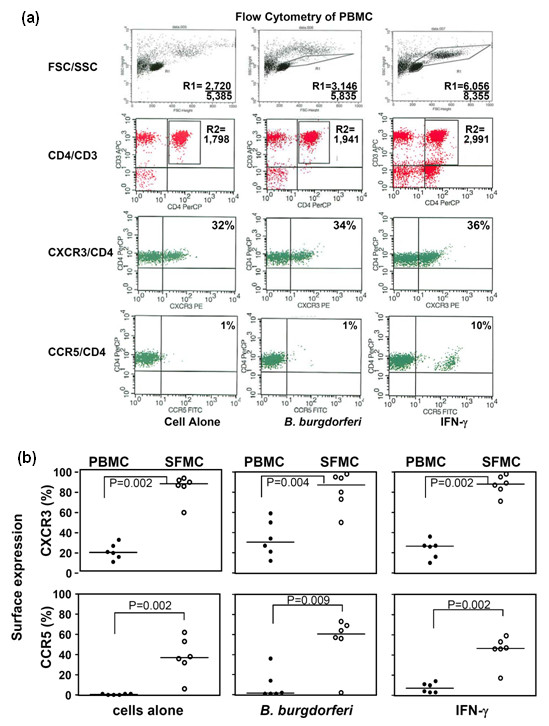
**The expression of the chemokine receptors CXCR3 and CCR5 on unstimulated or *Borrelia burgdorferi*- or interferon-gamma (IFN-γ)-stimulated peripheral blood mononuclear cells (PBMCs) or synovial fluid mononuclear cells (SFMCs) from six patients with Lyme arthritis**. **(a) **The flow cytometry data are shown for the PBMCs of one patient. After 5 days of incubation, nonadherent cells were resuspended, collected, and stained with antibodies. A forward scatter/side scatter (FSC/SSC) gate (R1) was used to exclude dead cells, and a CD3/CD4 gate (R2) was used to further restrict analysis to T cells. The number of events in each gate per number of events collected or the percentage of gated cells positive for CXCR3 or CCR5 is indicated on the histograms. **(b) **The percentages of T cells that were brighter than the isotype control are shown. The horizontal bars represent the median values. Significant differences between PBMCs and SFMCs are shown in brackets.

CXCR3, the receptor for CXCL9 and CXCL10 on T cells, and CCR5, the receptor for CCL4 on monocytes, dendritic cells, and some T cells, are preferentially expressed in T_H_1-like immune responses [7-9]. In 6 of the 11 study patients in whom concomitant PBMCs and SFMCs were available, the expression of these receptors on T cells was determined by flow cytometry (Figure 4a). In these patients, the percentage of unstimulated T cells expressing CXCR3 or CCR5 was significantly greater in SFMCs than PBMCs (Figure 4b). With PBMCs, *B. burgdorferi *or IFN-γ appeared to stimulate greater expression of CXCR3 or CCR5 compared with unstimulated cells. With SFMCs, CCR5 expression tended to increase with both stimuli, but the high expression of CXCR3 was rarely modulated further by either stimulus. However, none of the differences between stimulated and unstimulated cells was statistically significant.

## Discussion

In an effort to understand how *B. burgdorferi *stimulates cells of monocyte lineage to secrete chemokines that are prominent in the joint fluid of patients with Lyme arthritis, PBMCs, CD14^+ ^monocytes/macrophages from PBMCs, and SFMCs from patients were incubated with *B. burgdorferi *or IFN-γ or both, and the levels of these chemokines were measured in culture supernatants. *B. burgdorferi *directly stimulated CD14^+ ^cells to secrete CCL4, a chemoattractant for monocytes and some T cells [8]. In addition, we recently demonstrated that *B. burgdorferi *induces macrophages directly to secrete CXCL8, a neutrophil chemoattractant, and CCL3, a chemoattractant for monocytes, natural killer (NK) cells, and T cells [20]. These chemokines appear to be important in innate immune responses to *B. burgdorferi *[14]. In contrast, *B. burgdorferi *and IFN-γ were required to stimulate CD14^+ ^monocytes/macrophages to produce CCL2, whereas IFN-γ alone induced them to secrete CXCL9 and CXCL10. These chemokines are necessary for adaptive immune responses to the spirochete [14].

In this study, the efficiency of separation of CD14^+ ^cells from PBMCs was approximately 80% to 90%. However, it is unlikely that the CD14^- ^cells in this fraction had a functional role in our results. If significant numbers of T cells were still present in the CD14^+ ^fraction, we would have expected *B. burgdorferi *alone to have induced some production of CCL2, CXCL9, and CXCL10, as was the case with PBMCs. Instead, the levels of these chemokines were undetectable in *B. burgdorferi*-stimulated, CD14^+ ^cell cultures. Although dendritic cells can be generated from monocytes *in vitro *by culture with GM-CSF (granulocyte-macrophage colony-stimulating factor) and interleukin-4 [21], these conditions were not used here. Therefore, it is also unlikely that dendritic cells played a significant role in our results. We concluded that *B. burgdorferi *did not induce CD14^+ ^monocytes/macrophages directly to secrete CCL2, CXCL9, and CXCL10; other factors, likely including IFN-γ, were required from other intermediate cells that were present in PBMCs.

What other cells could be a source of IFN-γ or other factors leading to induction of IFN-γ-inducible chemokines? It was previously shown, using PBMCs from normal human donors, that *B. burgdorferi *stimulation of dendritic cells induced NK cells to secrete IFN-γ [22]. Moreover, in murine *B. burgdorferi *infection, CD1 d presentation of a borrelial glycolipid to NK T cells was important in the early innate immune response, possibly as a source of IFN-γ [23], and CD1d^-/- ^mice did not control the infection as well as their wild-type counterparts [24]. In murine cytomegalovirus infection [25] and in *Toxoplasma gondii *infection [26], early release of CCL3 and CCL4 from APCs is vital for the influx of NK cells into affected tissues, and these cells are an important source of IFN-γ, which induces macrophages and dendritic cells to secrete CXCL9 and CXCL10 [27]. Thus, NK cells or NK T cells or both are likely sources for the initial secretion of IFN-γ in Lyme disease. However, it is not yet known whether these cells play a role in joint inflammation in patients with Lyme arthritis.

Although we focused here on the role of IFN-γ in stimulating CD14^+ ^monocytes/macrophages to secrete CXCL9 and CXCL10, other factors and cell types may be involved in the actual infection. For example, type I IFNs, particularly IFN-α, may also have a role in inducing the secretion of these chemokines [28,29]. Moreover, under appropriate conditions, neutrophils secrete a number of cytokines and chemokines, including type I IFNs [30,31]. Thus, in human Lyme arthritis, it is possible that multiple cell types produce multiple IFNs, which induce cells of monocyte lineage to secrete CXCL9 and CXCL10, thereby leading to the recruitment of T effector cells to the joint.

It is not yet known how *B. burgdorferi *stimulates monocytes/macrophages to secrete CCL4, nor is it clear how spirochetes in the presence of IFN-γ induce CCL2 expression. *B. burgdorferi *is known to activate monocytes/macrophages through multiple types of receptors [32-36]. The spirochete is composed of many lipoproteins, which presumably stimulate macrophages through the Toll-like receptor-1 (TLR1)/TLR2 heterodimer [32,33]. However, live *B. burgdorferi*, as used here, induces monocyte activation by both TLR2-dependent and TLR2-independent pathways [34-36]. Moreover, TLR2 ligation is not involved in the secretion of the IFN-γ-inducible chemokines CCL2, CXCL9, and CXCL10 [37].

Without stimulation, the percentage of cells expressing CXCR3 and CCR5 was significantly greater in SFMCs than PBMCs, confirming that T_H_1 effector cells were recruited to the site of a T_H_1 inflammatory response in affected joints [12,13]. Consistent with this result, cells expressing CXCR3 and CCR5 were found previously in synovial tissue in patients with Lyme arthritis or rheumatoid arthritis [14,38]. In comparison with unstimulated cells, there were no significant changes in the expression of these receptors after stimulation with *B. burgdorferi *or IFN-γ. Moreover, although SFMCs probably contained a larger percentage of activated cells, PBMCs and SFMCs responded similarly to *B. burgdorferi *and IFN-γ in cell cultures.

## Conclusions

*B. burgdorferi *activates monocytes/macrophages directly to secrete a number of chemokines, including CCL4, which are important in innate immune responses both early and late in Lyme disease. At the same time, spirochetes stimulate other intermediate cells, possibly NK or NK T cells, to produce IFN-γ or other factors, which then induce innate immune cells to secrete CCL2, CXCL9, and CXCL10, thereby serving as a bridge between innate and adaptive immune responses. We conclude that *B. burgdorferi *stimulates monocytes/macrophages directly and indirectly to guide innate and adaptive immune responses in patients with Lyme arthritis.

## Abbreviations

APC: antigen-presenting cell; EM: erythema migrans; FACS: fluorescence-activated cell sorting; IFN-γ: interferon-gamma; i.v.: intravenous; MACS: magnetic-activated cell sorting; MOI: multiplicity of infection; NK: natural killer; PBMC: peripheral blood mononuclear cell; PBS: phosphate-buffered saline; PCR: polymerase chain reaction; SFMC: synovial fluid mononuclear cell; T_H_1: T helper 1; TLR: Toll-like receptor.

## Competing interests

The authors declare that they have no competing interests.

## Authors' contributions

JJS and ACS had full access to all of the data in the study and share responsibility for the integrity of the data and the accuracy of the data analysis; they were involved in the acquisition and analysis of the data and helped to design the study, to prepare the manuscript, and to perform the statistical analyses. LJG had full access to all of the data in the study and shares responsibility for the integrity of the data and the accuracy of the data analysis and helped to design the study, to analyze the data, and to prepare the manuscript. KS was involved in the acquisition of the data and helped to analyze the data and to prepare the manuscript. ADL helped to prepare the manuscript. All authors read and approved the final manuscript.

## Acknowledgements

We thank Gail McHugh for help with the collection of patient samples and Colleen Squires for help with preparation of the manuscript. This research was supported by the National Institutes of Health (AR-20358); the English, Bonter, Mitchell Foundation; the Lyme/Arthritis Research Fund at Massachusetts General Hospital; and the Eshe Fund. JJS and KS received support from scholarships from the Walter J. and Lille A. Berbecker Foundation for the study of Lyme disease, and JJS received support from a National Institutes of Health training grant (AR-007258).
